# Supplementary material for: Immunogenetic Predisposition to SARS-CoV-2 Infection
Source: Biology (Basel). 2022 Dec 25;12(1):37. doi: 10.3390/biology12010037 (PMC9855425; doi:10.3390/biology12010037)
Supplement: Supplementary file 1 [file biology-12-00037-s001.zip › biology-2101839-supplementary.pdf]

Immunogenetic predisposition to SARS-CoV-2 infection

Claudia Lehmann, Henry Loeffler-Wirth, Vera Balz, Juergen Enzmann, Ramona Landgraf, Nicole Lakowa, Thomas Gruenewald, Johannes C. Fischer and Ilias Doxiadis

Supplementary Materials

Table S1. Characteristics of primers used in the NGS test system. Numbering is referenced to Genome build GRCh37.1 / hg19.

Blood Group Analysis					
Blood Group System	Gene	Target	Chromosome	Amplified Region	Amplicon Size (bp)
ABO	*ABO*	Exon 1	9	136,150,360..136,150,792	433
		Exon 2		136,137,169..136,137,640	473
		Exon 3		136,136,687..136,137,164	478
		Exon 4		136,135,052..136,135,503	452
		Exon 5		136,133,318..136,133,692	375
		Exon 6		136,132,640..136,133,028	389
		Exon 7 (5′-part)		136,131,265..136,131,768	504
		Exon 7 (3′-part)		136,130,907..136,131,370	464
MNS	*GYPA*	Exon 2	4	145,041,554..145,041,915	371
		Exon 3		145,040,595..145,040,984	389
		Exon 4		145,039,569..145,040,051	482
		Exon 5		145,037,862..145,038,303	441
	GYPB	Exon 2		144,922,248..144,922,611	363
		Exon 3		144,921,292..144,921,678	386
		Exon 4		144,920,260..144,920,748	488
		Exon 5		144,918,548..144,918,989	441
	GYPE	Exon 2		144,801,474..144,801,836	363
		Exon 3		144,800,519..144,800,905	387
		Exon 4		144,799,488..144,799,974	487
		Exon 5		144,918,548..144,918,989	442
P1PK	*A4GALT*	Promoter	22	43,113,665..43,114,050	386
		Exon 3		43,089,693..43,090,192	500
RH	*RHCE*	Exon 1	1	25,747,087..25,747,462	375
		Exon 2		25,734,981..25,735,422	441
		Exon 3		25,728,976..25,729,399	423
		Exon 4		25,718,433..25,718,818	385
		Exon 5		25,714,146..25,714,461	315
		Exon 6		25,715,409..25,715,772	363

		Exon 7		25,712,081..25,712,376	295
		Exon 8		25,701,673..25,702,183	510
		Exon 9		25,696,742..25,697,196	454
		Exon 10		25,688,873..25,689,316	444
	RHD	Exon 1		25,598,854..25,599,229	376
		Exon 2		25,611,002..25,611,442	414
		Exon 3		25,616,959..25,617,393	435
		Exon 4		25,627,252..25,627,636	385
		Exon 5		25,627,956..25,628,271	316
		Exon 6		25,629,645..25,630,027	383
		Exon 7		25,633,046..25,633,341	296
		Exon 8		25,643,227..25,643,737	511
		Exon 9		25,648,217..25,648,669	453
		Exon 10		25,655,117..25,655,557	441
LU	*BCAM*	Exon 3 + 4	19	45,315,383..45,315,826	444
		Exon 5 + 6		45,316,448..45,316,929	482
		Exon 9 + 10		45,321,712..45,322,205	494
		Exon 11		45,322,159..45,322,646	488
		Exon 12 + 13		45,322,549..45,323,036	488
		Exon 14		45,323,885..45,324,210	326
KEL	*KEL*	Exon 6	7	142,654,699..142,655,136	438
		Exon 7 + 8		142,651,220..142,651,653	434
		Exon 13		142,641,163..142,641,657	495
		Exon 15 + 16		142,640,296..142,640,789	494
		Exon 17		142,639,813..142,640,248	436
		Exon 18		142,639,298..142,639,705	408
		Exon 19		142,638,042..142,638,536	495
LE	*FUT3*	Exon 3 (5′-part)	19	5,844,506..5,844,994	489
		Exon 3 (center part)		5,844,091..5,844,582	492
		Exon 3 (3′-part)		5,843,719..5,844,208	490
FY	*ACKR1*	5′-UTR + Exon 1	1	159,174,621..159,175,080	460
		Exon 2 (5′-part)		159,175,132..159,175,623	492
		Exon 2 (center part)		159,175,557..159,176,044	488
		Exon 2 (3′-part)		159,175,842..159,176,329	488
JK	*SLC14A1*	Exon 3	18	43,310,204..43,310,640	437
		Exon 4		43,310,772..43,311,228	457
		Exon 5		43,314,188..43,314568	381
		Exon 6		43,316,359..43,316,727	369
		Exon 7		43,318,864..43,319,352	489
		Exon 8		43,319,333..43,319,793	461
		Exon 9		43,328,196..43,328,591	396

		Exon 10		43,329,608..43,329,971	364
DI	*SLC4A1*	Exon 16	17	42,331,735..42,332,220	486
		Exon 18 + 19		42,328,399..42,328,870	472
YT	*ACHE*	Exon 2 (3′-part)	7	100,490,697..100,491,187	491
DO	*ART4*	Exon 2 (5′-part)	12	14,993,734..14,994,149	416
		Exon 2 (3′-part)		14,993,335..14,993,806	472
CO	*AQP1*	Exon 1 (5′-part)	7	30,951,346..30,951,811	466
		Exon 1 (3′-part)		30,951,599..30,952,042	444
H	*FUT1*	Exon 4 (5′-part)	19	49,254,122..49,254,606	485
		Exon 4 (center part)		49,253,825..49,254,310	486
		Exon 4 (3′-part)		49,253,386..49,253,878	493
	FUT2	Exon 2 (5′-part)	19	49,206,121..49,206,613	493
		Exon 2 (center part)		49,206,496..49,206,968	473
		Exon 2 (3′-part)		49,206,911..49,207,379	469
GE	*GYPC*	Exon 2	2	127,447,552..127,447,948	397
		Exon 3		127,451,277..127,451,740	464
CROM	*CD55*	Exon 2	1	207,495,575..207,496,056	482
		Exon 6		207,504,312..207,504,807	496
KN	*CR1*	Exon 26	1	207,760,472..207,760,944	473
		Exon 29		207,782,603..207,783,071	469
IN	*CD44*	Exon 2	11	35,198,016..35,198,509	494
JR	*ABCG2*	Exon 2	4	89,060,870..89,061,363	494
		Exon 4		89,052,838..89,053,229	392
		Exon 5		89,052,155..89,052,628	474
		Exon 7		89,039,032..89,039,525	494
		Exon 9		89,034,316..89,034,792	477
		Exon 13		89,018,491..89,018,904	414
		Exon 16		89,013,275..89,013,636	362
LAN	*ABCB6*	Exon 3	2	220,081,281..220,081,759	479
		Exon 5		220,080,686..220,081,079	394
		Exon 6		220,079,574..220,079,951	378
		Exon 7		220,079,072..220,079,541	470
		Exon 9		220,078,441..220,078,835	395
		Exon 10 + 11		220,078,110..220,078,485	376
		Exon 12 + 13		220,077,671..220,078,160	490
VEL	*SMIM1*	Exon 3	1	3,691,784..3,692,199	416
		Exon 4		3,692,346..3,692,722	377
AT	*SLC29A1*	Exon 6	6	44,197,953..44,198,428	476
		Exon 12		44,200,392..44,200,878	487

Platelet Antigen Analysis				
Gene	Target	Chromosome	Amplified Region	Amplicon Size (bp)
ITGB3	Exon 3	17	45,360,632..45,361,068	437
	Exon 4		45,361,730..45,362,156	427
GP1BA	Exon 2	17	4,836,200..4,836,693	494
ITG2A	Exon 13	5	52,358,675..52,358,987	313
ITBA2B	Exon 22	17	42,452,639..42,453,109	471
CD109	Exon 19	6	74,493,384..74,493,817	434

Summarized Alleles

In addition, for blood group genes for which not the entire transcript is analyzed, we introduced the term "eGroup", e.g. the genotyping results carry an "E001 to E004"-tag. Here, similar to the G-group definition previously known for molecular genotyping results for Human Leukocyte Antigens (HLA), all alleles are summarized which share identical genomic sequence for all analyzed parts.

Table S2: Nomenclature of non-referenced alleles

Example 1:

BG-System or gene[1]	Divider 1[2]	Most similar ISBT allele[3]	Divider 2[4]	Amino acid change[5]	Divider 3[6]	Nucleotide change[7]
DO	*	01	.	D86H	_	378C_T

Example 2:

BG-System or gene[1]	Divider 1[2]	Most similar ISBT allele[3]	Divider 2[4]	Nucleotide change[7]
ABO	*	A1.01	.	E3-10C_T

Example 3:

BG-System or gene[1]	Divider 1[2]	Most similar ISBT allele[3]	Divider 2[4]	Pseudoexon[8]	Divider 4[9]	Nucleotide change[7]
GYPB	*	04	.	E3	.	41T_C

[1] Depending on the ISBT nomenclature the BG-system name or the gene name is used.

[2] Divider 1 (*) indicates a result that was obtained using molecular typing methods.

[3] The most similar ISBT allele as published by the ISBT working parties (http://www.isbtweb.org/working-parties/red-cell-immunogenetics-and-blood-group-terminology/).

[4] Divider 2 is always a ".". It indicates that additional amino acid changes or nucleotide changes are present.

[5] Amino acid changes are listed in consecutive order. They are termed in one-letter-code with the position of amino acid in the middle.

[6] Divider 3 "_" is used to separate additional amino acid changes or nucleotide changes in the cDNA or changes which are present in intron.

[7] Nucleotide changes, which do not result in an amino acid change are termed as nucleotide position in cDNA immediately followed by wild type nucleotide and changed nucleotide separated by "_". In case of changes in intron regions, the nearest exon is given followed by distance to the exon in counts of nucleotide.

+, Nucleotide change is downstream of exon.

-, Nucleotide change is upstream of exon.

[8] Divider 4 "." indicates that the following nucleotide change is present in a pseudoexon (valid for GYPB and GYPE, only).

Figure S1: Frequencies of classical HLA loci in the Saxonian population. The results are depicted in the highest to lower number of individuals per allele.

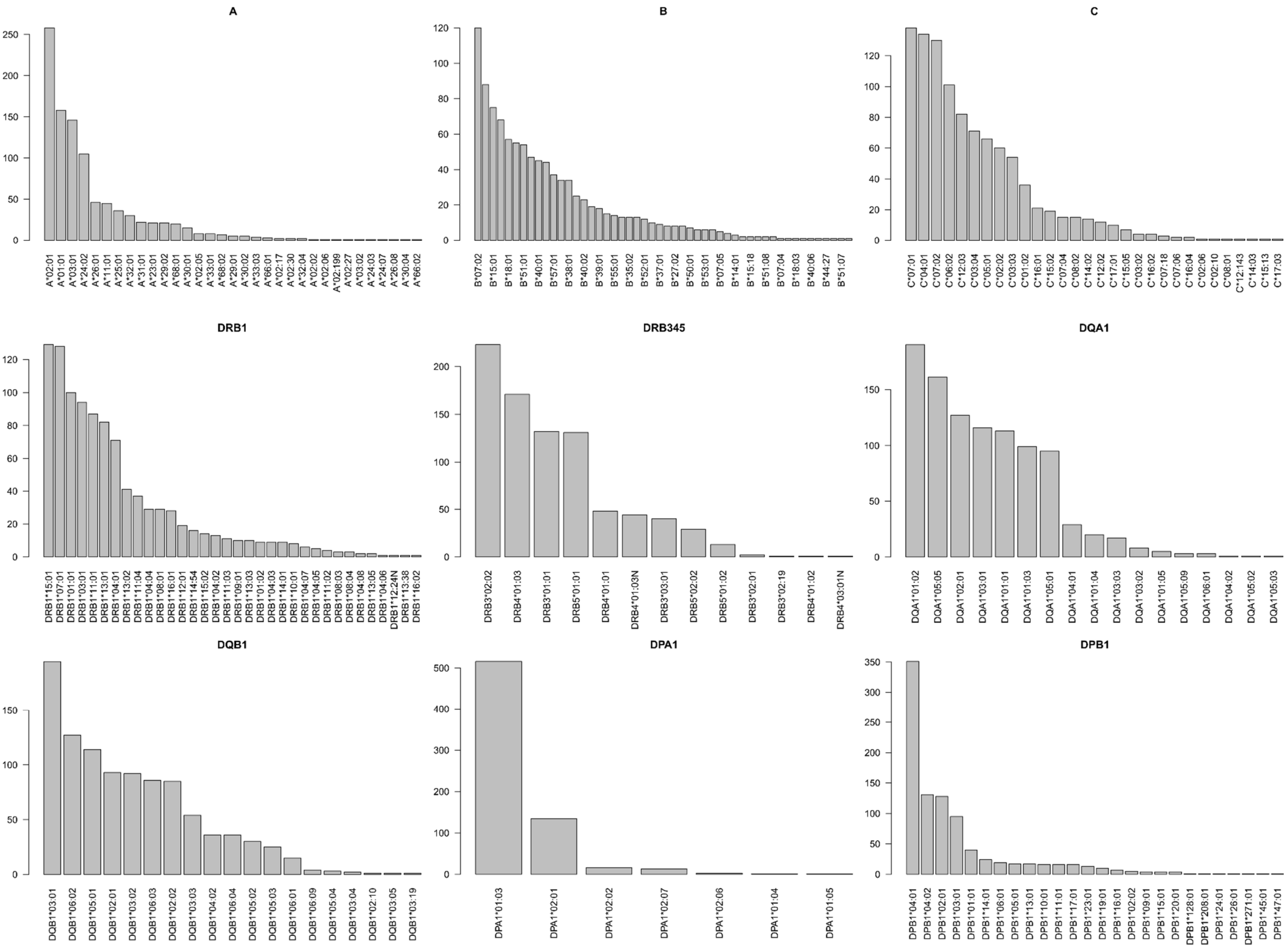

Figure S2: Frequencies of non-classical HLA loci in the Saxonian population, defined in 134 unrelated individuals of the present study. No selection was done. The results are depicted in the highest to lower number of individuals per allele.

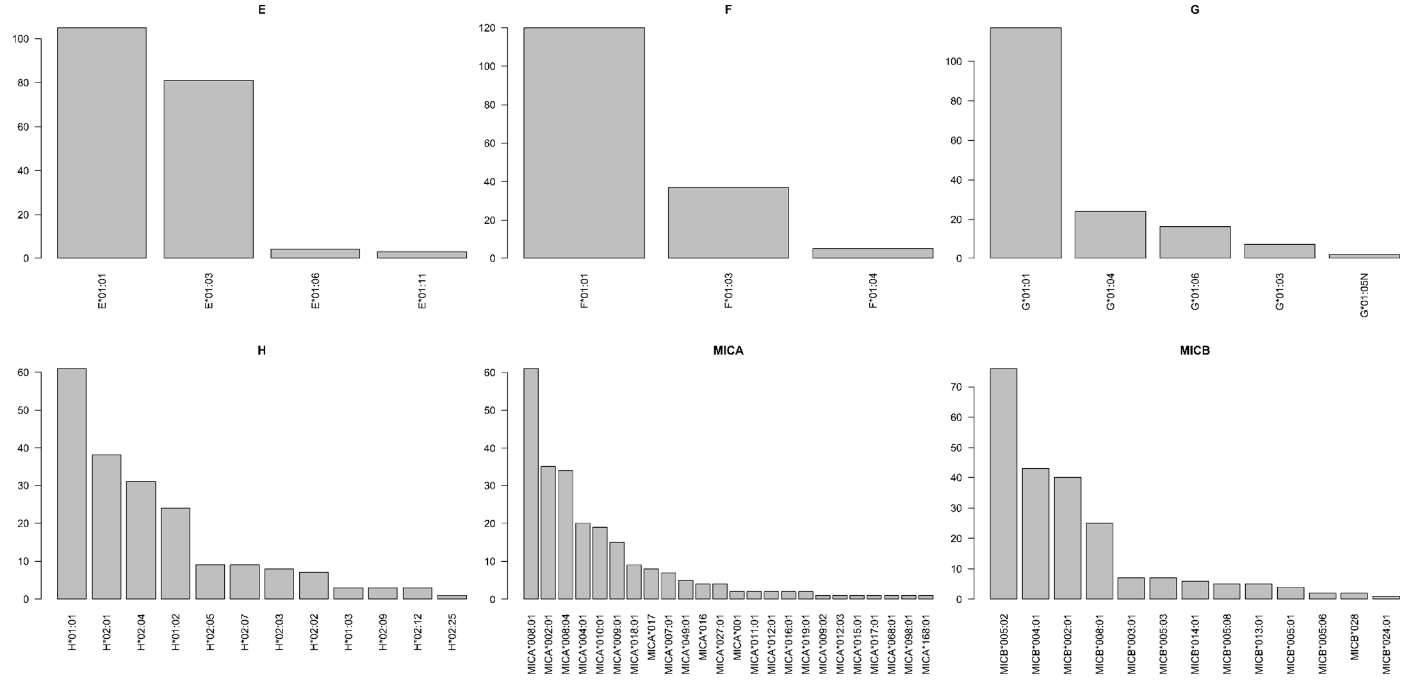

Figure S3: Evaluation of HLA class II alleles within the groups of PCR positive and PCR negative individuals. t-statistics of positive PCR proportion against the overall positive rate provides p-values, which were corrected for multiple testing using the Bonferroni method. The horizontal line indicates a corrected p-value of 0.05.

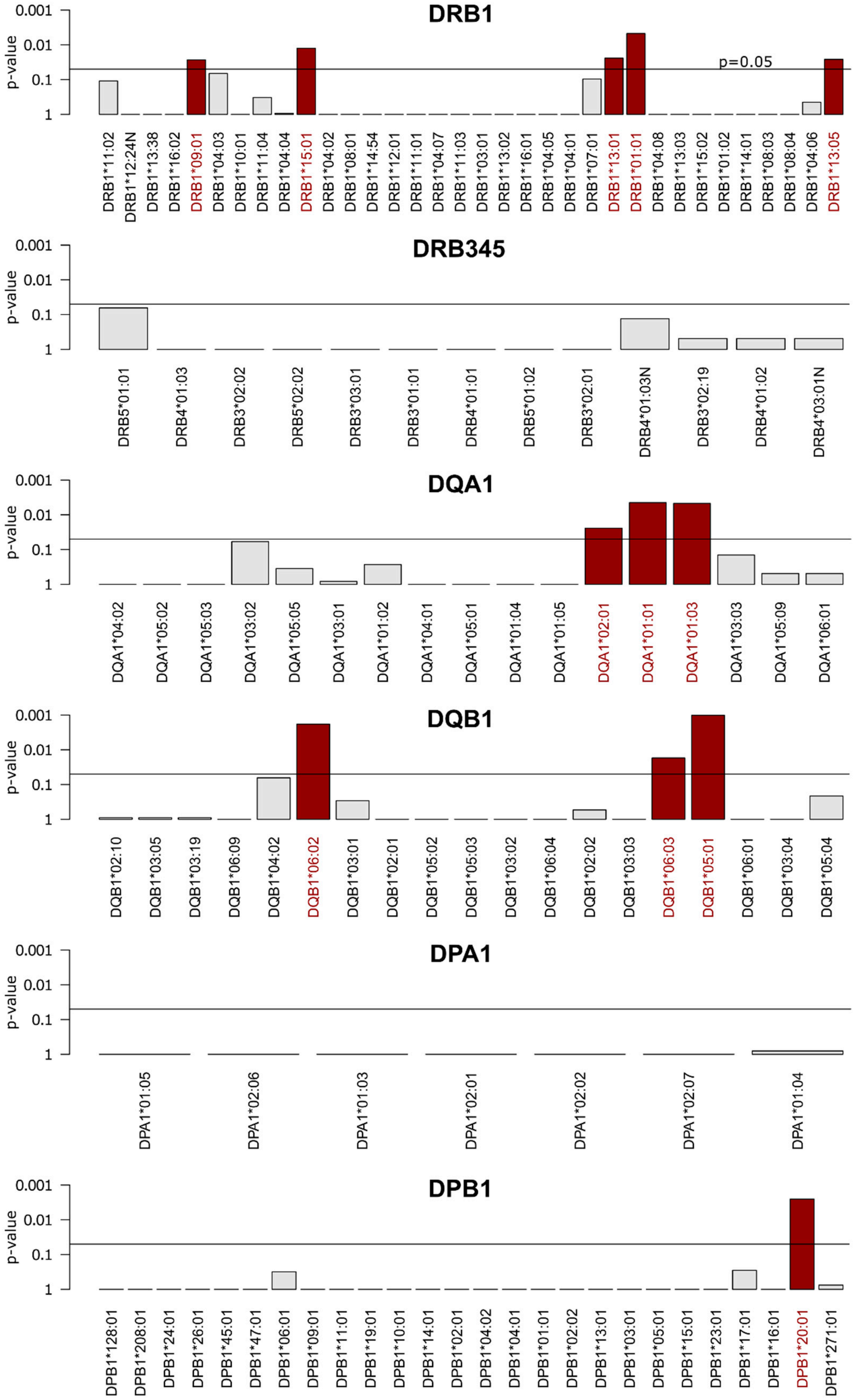

Figure S4: Frequencies of blood group and HPA antigens in the Saxonian population

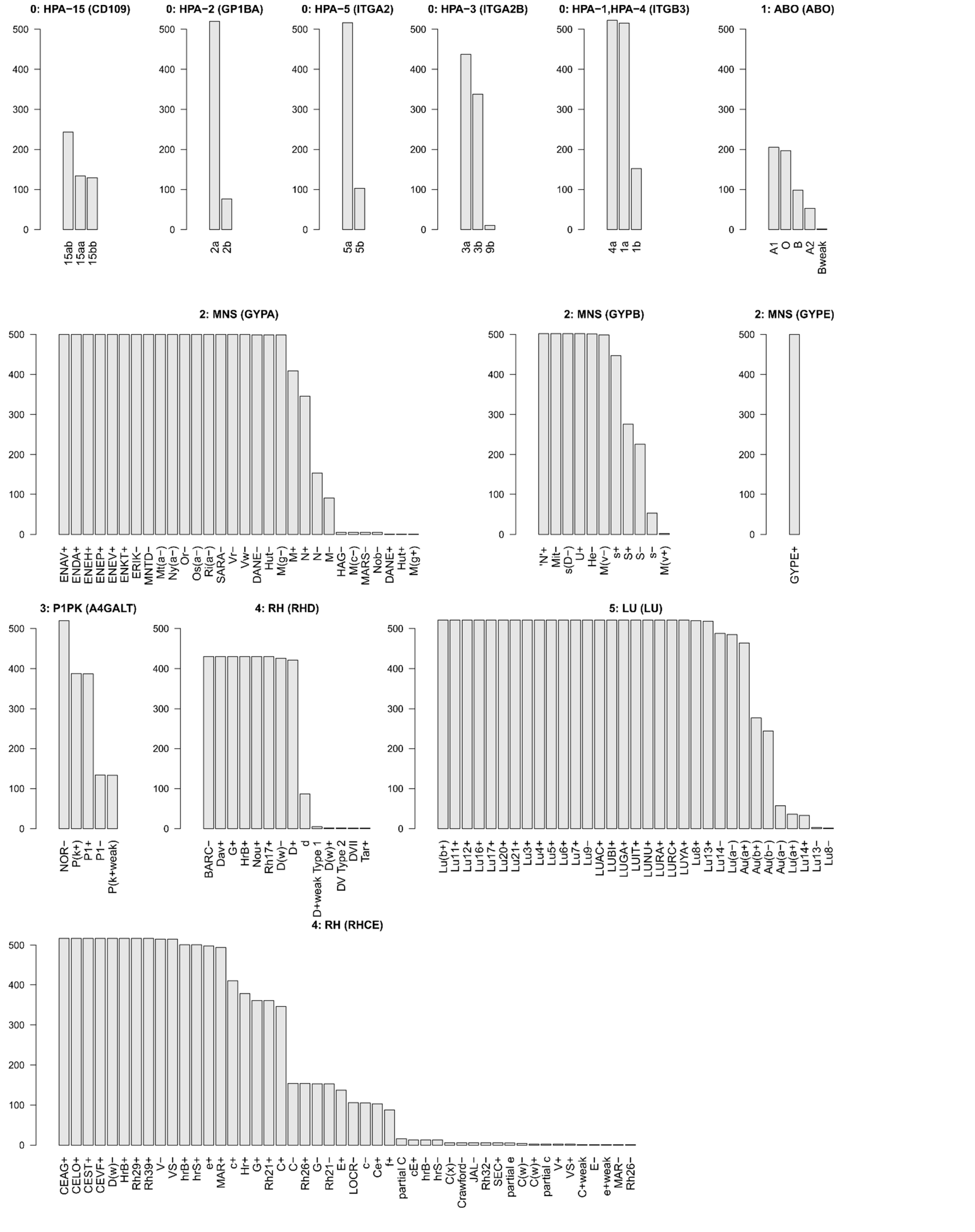

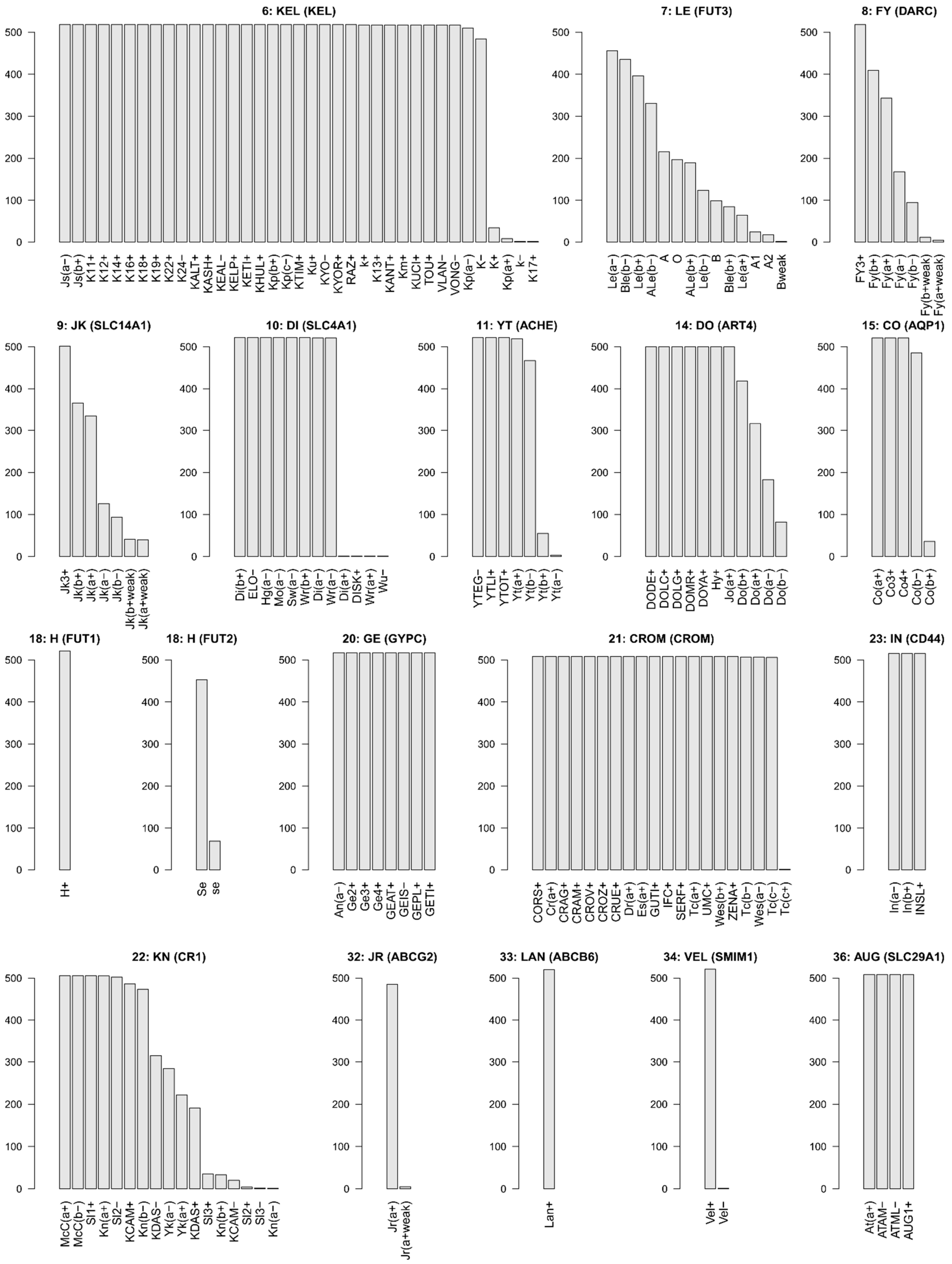
6: KEL (KEL)
Js(a−) Js(b+) K11+ K12+ K14+ K16+ K18+ K19+ K22+ K24− KALT+ KASH+ KEAL− KELP+ KETI+ KHUL+ Kp(b+) Kp(c−) KTIM+ Ku+ KYO− KYOR+ RAZ+ k+ K13+ KANT+ Km+ KUCI+ TOU+ VLAN− VONG− Kp(a−) K− K+ Kp(a+) k− K17+
7: LE (FUT3)
Le(a−) Ble(b−) Le(b+) ALe(b−) A O ALe(b+) Le(b−) B Ble(b+) Le(a+) A1 A2 Bweak
8: FY (DARC)
FY3+ Fy(b+) Fy(a+) Fy(a−) Fy(b−) Fy(b+weak) Fy(a+weak)
9: JK (SLC14A1)
Jk3+ Jk(b+) Jk(a+) Jk(a−) Jk(b−) Jk(b+weak) Jk(a+weak)
10: DI (SLC4A1)
Di(b+) ELO− Hg(a−) Mo(a−) Sw(a−) Wr(b+) Di(a−) Wr(a−) Di(a+) DISK+ Wr(a+) Wu−
11: YT (ACHE)
YTEG− YTLI+ YTOT+ Yt(a+) Yt(b−) Yt(b+) Yt(a−)
14: DO (ART4)
DODE+ DOLC+ DOLG+ DOMR+ DOYA+ Hy+ Jo(a+) Do(b+) Do(a+) Do(a−) Do(b−)
15: CO (AQP1)
Co(a+) Co3+ Co4+ Co(b−) Co(b+)
18: H (FUT1)
H+
18: H (FUT2)
Se se
20: GE (GYPC)
An(a−) Ge2+ Ge3+ Ge4+ GEAT+ GEIS− GEPL+ GETI+
21: CROM (CROM)
CORS+ Cr(a+) CRAG+ CRAM+ CROV+ CROZ+ CRUE+ Dr(a+) Es(a+) GUTI+ IFC+ SERF+ Tc(a+) UMC+ Wes(b+) ZENA+ Tc(b−) Wes(a−) Tc(c−) Tc(c+)
23: IN (CD44)
In(a−) In(b+) INSL+
22: KN (CR1)
McC(a+) McC(b−) Sl1+ Kn(a+) Sl2− KCAM+ Kn(b−) KDAS− Yk(a−) Yk(a+) KDAS+ Sl3+ Kn(b+) KCAM− Sl2+ Sl3− Kn(a−)
32: JR (ABCG2)
Jr(a+) Jr(a+weak)
33: LAN (ABCB6)
Lan+
34: VEL (SMIM1)
Vel+ Vel−
36: AUG (SLC29A1)
At(a+) ATML− ATML+ AUG1+
0 100 200 300 400 500

Figure S5: t-test of positive PCR proportion against the overall positive rate provides p-values, which were corrected for multiple testing using the Bonferroni method. The horizontal line indicates a corrected p-value of 0.05. Blood groups with corrected p-value<0.05 are highlighted in red

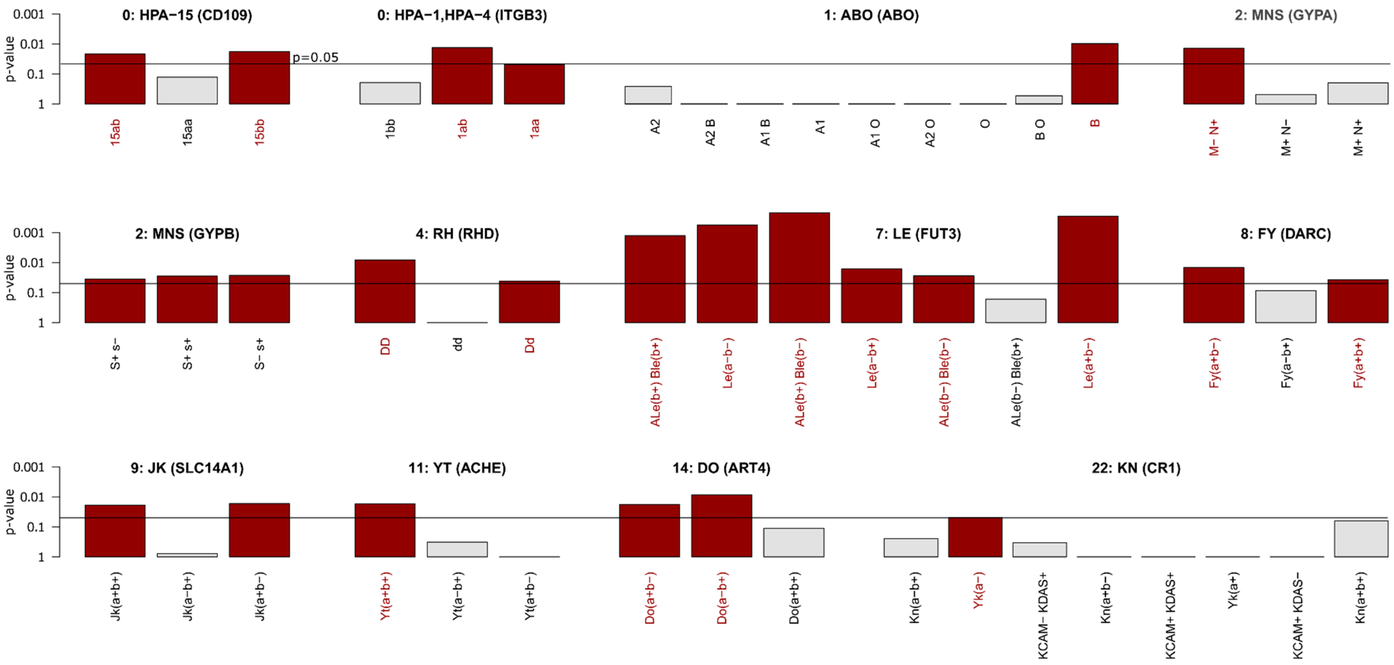

Figure S6: Blood groups with no enrichment in both groups (COVID-19 infected and non-infected group): Percent of persons with positive PCR stratified by the alleles. Black and gray bars show the proportion of PCR positive and negative persons in a particular allele, respectively. The overall positive PCR rate of 62% is indicated by the horizontal line. Below: t-statistics of positive PCR proportion against the overall positive rate provides p-values, which were corrected for multiple testing using the Bonferroni method. The horizontal line indicates a corrected p-value of 0.05.

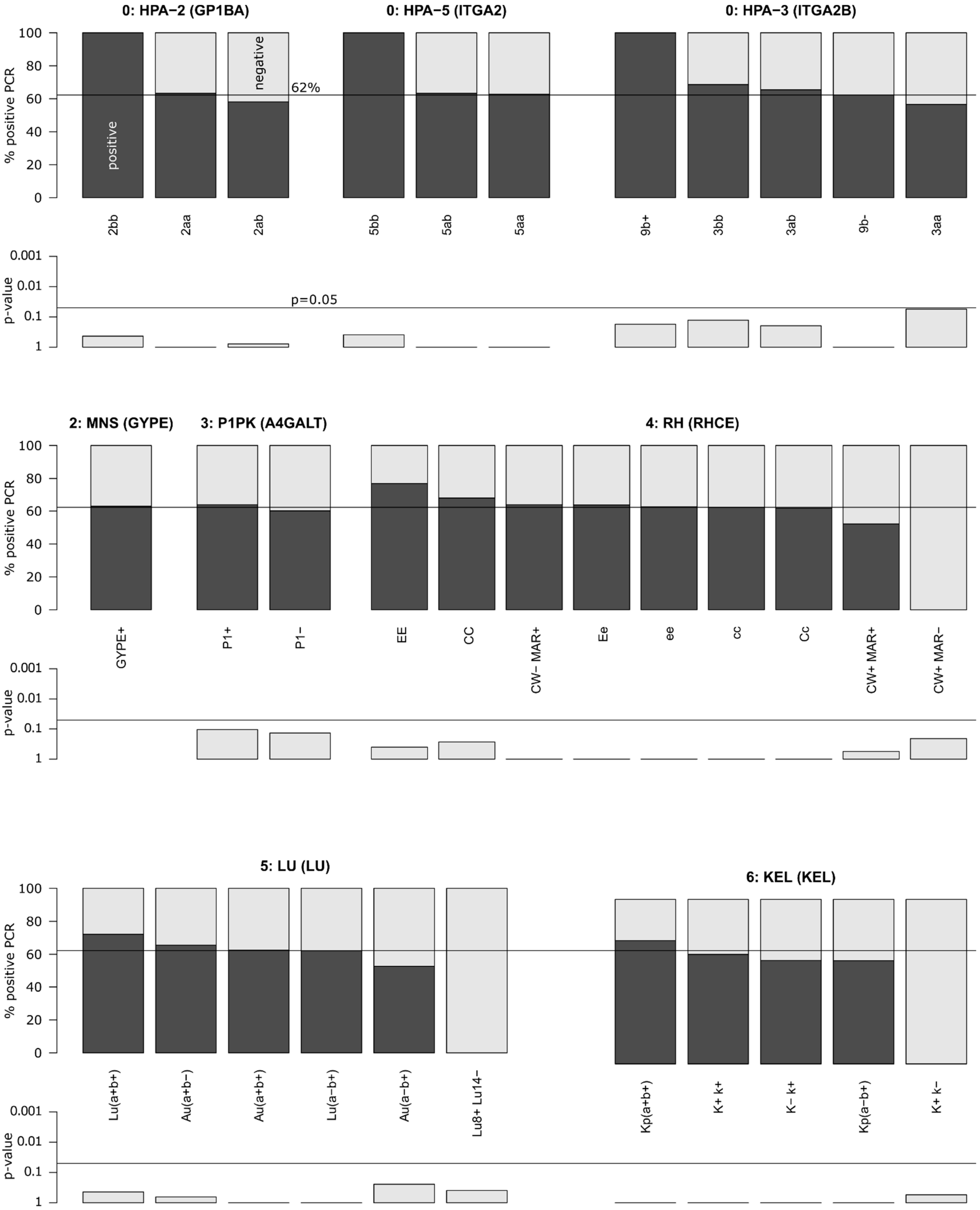

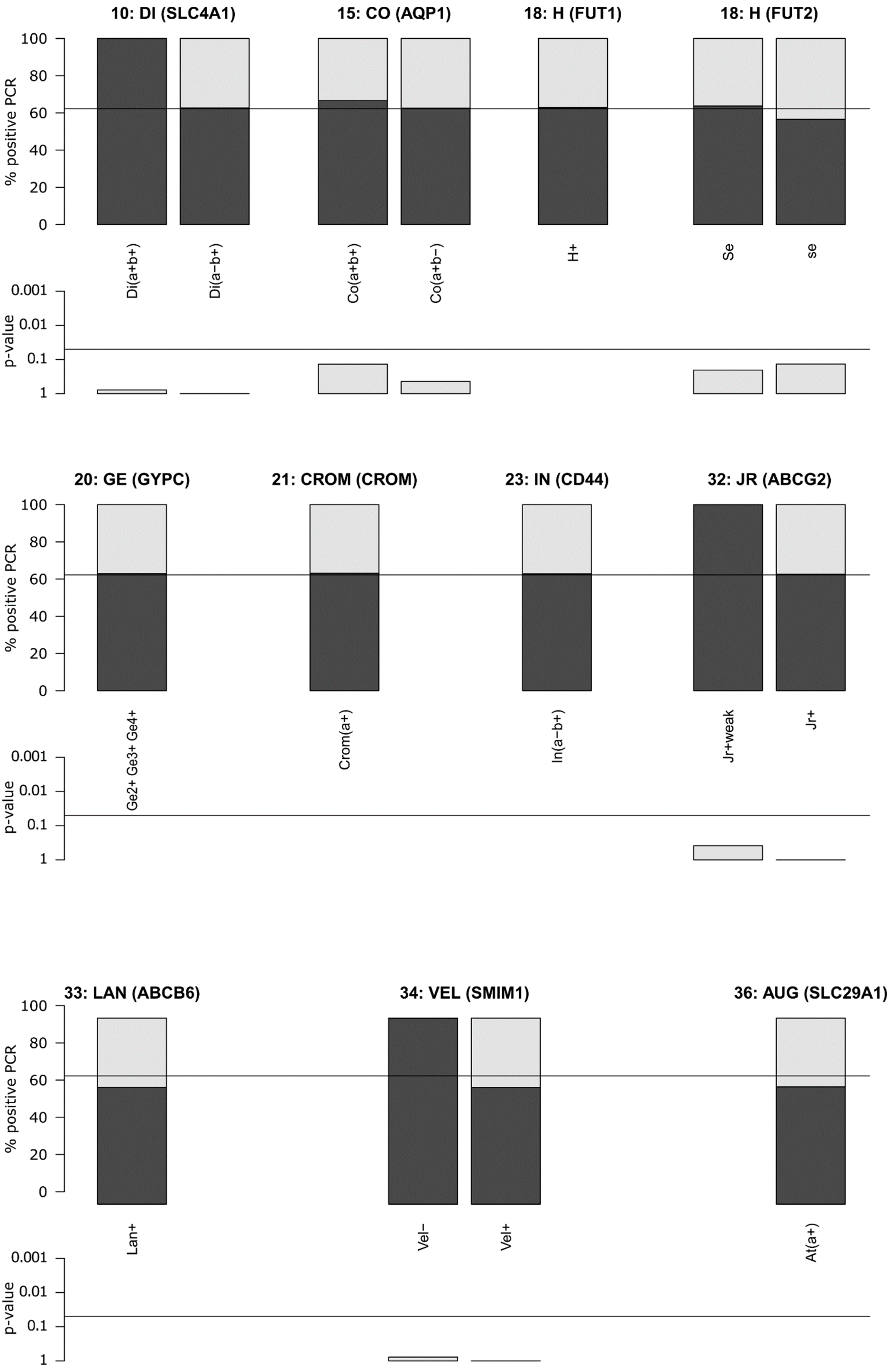

10: DI (SLC4A1)
15: CO (AQP1)
18: H (FUT1)
18: H (FUT2)
% positive PCR
p-value
Di(a+b+)
Di(a−b+)
Co(a+b+)
Co(a+b−)
H+
Se
se
20: GE (GYPC)
21: CROM (CROM)
23: IN (CD44)
32: JR (ABCG2)
Ge2+ Ge3+ Ge4+
Crom(a+)
In(a−b+)
Jr+weak
Jr+
33: LAN (ABCB6)
34: VEL (SMIM1)
36: AUG (SLC29A1)
Lan+
Vel−
Vel+
At(a+)
